# *Viscum album* L. Extracts Protects HeLa Cells against Nuclear and Mitochondrial DNA Damage

**DOI:** 10.1155/2012/958740

**Published:** 2012-08-16

**Authors:** Evren Önay-Uçar, Özlem Erol, Başak Kandemir, Elif Mertoğlu, Ali Karagöz, Nazlı Arda

**Affiliations:** ^1^Department of Molecular Biology and Genetics, Faculty of Science, Istanbul University, Vezneciler, 34118 Istanbul, Turkey; ^2^Department of Biology, Faculty of Sciences and Arts, Çanakkale Onsekiz Mart University, Terzioglu Campus, 17020 Çanakkale, Turkey; ^3^Department of Genetics and Bioengineering, Molecular Neurobiology Laboratory, Yeditepe University, 26 Agustos Campus Kayisdagi Street, Kayisdagi, 81120 Istanbul, Turkey; ^4^Institute of Forensic Sciences, Istanbul University, Cerrahpaşa Campus, 34303 Istanbul, Turkey

## Abstract

*Viscum album* L. is a semiparasitic plant grown on trees and widely used for the treatment of many diseases in traditional and complementary therapy. It is well known that some activities of *Viscum album* extracts are varied depending on the host trees, such as antioxidant, apoptosis-inducing, anticancer activities of the plant. The aim of the present study is to examine the comparative effects of methanolic extracts of *V. album* grown on three different host trees (locust tree, lime tree, and hedge maple tree) on H_2_O_2_-induced DNA damage in HeLa cells. Oxidative damage in mitochondrial DNA and two nuclear regions was assessed by QPCR assay. The cells were pretreated with methanolic extracts (10 **μ**g/mL) for 48 h, followed by the treatment with 750 **μ**M H_2_O_2_ for 1 hour. DNA damage was significantly induced by H_2_O_2_ while it was inhibited by *V. album* extracts. All extracts completely protected against nuclear DNA damage. While the extract from lime tree or white locust tree entirely inhibited mitochondrial DNA damage, that from hedge maple tree inhibited by only 50%. These results suggest that methanolic extracts of *V. album* can prevent oxidative DNA damage, and the activity is dependent on the host tree.

## 1. Introduction

Reactive oxygen species (ROS) which originate from a range of cellular processes, external factors, and/or various diseases can damage cellular components [1]. The enzymatic and nonenzymatic antioxidant defense systems are natural protectors against oxidative stress caused by ROS. However, these mechanisms cannot completely protect DNA against damage [2]. Although oxidative DNA damage can be repaired, unrepaired damage can accumulate in the cell. The most dramatic results of this accumulation are mutations and cell death [3]. Hence, oxidative DNA damage is an important factor for the aging process and age-related diseases, such as cancer [1, 4, 5]. 

*Viscum album* L. (mistletoe) is a semiparasitic perennial plant that grows on different host trees [6]. Different *Viscum album* extracts have been used in traditional medicine for the treatment of various diseases such as stroke, atherosclerosis, hypertension, and diabetes [7]. This plant has many biological activities such as anticancer, antiviral, antioxidant, apoptosis-inducing and immunomodulatory properties [8–15]. Although methanolic extract of *Viscum album* leaves is able to reduce the malondialdehyde (MDA) and reduce glutathione (GSH) levels on kidney and heart of streptozotocin-induced diabetic rats and has strong antioxidant activity [11–13], the effect on oxidative DNA damage has not been examined in HeLa cells. The main purpose of the present study is to investigate whether the methanolic extract of *Viscum album* protects nDNA and mtDNA against H_2_O_2_-induced oxidative stress in HeLa cells. 

## 2. Materials and Methods

### 2.1. Plant Materials

*Viscum album* L. plants grown on three different host trees (lime tree (*Tilia argentea* Desf. Ex DC, Tv), hedge maple tree (*Acer campestre *L. ssp. c*ampestre,* Av), and locust tree (*Robinia pseudoacacia* L., Rv)) were harvested from the Northern part of Istanbul in September 2006. The voucher specimen was identified and deposited in the Herbarium of Biology Department, Istanbul University, Istanbul, Turkey (Tv, ISTF 37486; Av, ISTF 37487; Rv, ISTF 37488). Fresh leaves of each sampling were picked and washed by tap water, followed by distilled water. After drying they were cut into small pieces, weighed, and used immediately or stored separately at −20°C until use. 

### 2.2. Preparation of Extracts

The extracts were prepared as described earlier [12]. Fresh leaves (20 g) were macerated in methanol (160 mL) in an incubatory shaker (150 rev/min, 25°C) for 24 hours. After removing the plant residues by filtration, each filtrate was evaporated to dryness under vacuum, and the dried material was weighed. Crude extracts were dissolved in DMSO at a concentration of 40 mg/mL and stored at −20°C until use. They were designated according to the trees where the plants were collected as Tv (*Tilia viscum*), Av (*Acer viscum*), and Rv (*Robinia viscum*). 

### 2.3. Cell Culture

Human HeLa cervical carcinoma cells were cultured in Eagle's Minimum Essential Medium (EMEM) supplemented with 10% (v/v) heat-inactivated fetal bovine serum and antibiotic-antimycotic mixture (penicillin (100 U/mL), streptomycin (100 *μ*g/mL), amphotericin B (0.25 *μ*g/mL)). Cells were seeded at a concentration of 10^5^ cells/mL and maintained at 37°C in an atmosphere with 5% CO_2_. *V. album* extract was added to the growth medium, after dissolving in DMSO at a final concentration not exceeding 0.5% (v/v), since DMSO is able to inhibit cell growth above this concentration (data not shown).

### 2.4. Cytotoxicity Test

The cytotoxic activity of the extracts was tested on HeLa cells by using the 3-(4,5-dimethylthiazol-2-yl)-2,5-diphenyltetrazolium bromide (MTT) assay based on the reduction of MTT to a colored formazan product by mitochondrial dehydrogenase, which is active only in living cells [16]. The stock solutions of the extracts were diluted with EMEM. Cells (10^5^ cells/mL) were seeded into each well of a 96-well plate, containing 200 *μ*L EMEM. After reaching confluence (24 h later), the cells were treated with increasing concentrations (1–500 *μ*g/mL) of *V. album* methanol extract diluted with EMEM for 48 h. To determine cytotoxic activity of H_2_O_2_ the cells were treated with different concentrations (0.05–5 mM) of H_2_O_2_ diluted with Hank's balanced salt solution (HBBS) for 1 h at the end of 72 h. In the next step, upper layers were discarded. After washing the adherent cells with phosphate buffer saline (PBS) to minimize the interference of upper layer residue, 10 *μ*L of MTT stock solution (5 mg/mL) was added to each well, and the plates were further incubated for 4 hours at 37°C. After formation of water insoluble purple formazan crystals, they were dissolved in 200 *μ*L of DMSO, and the resulting optical density was measured by a microplate reader (*μ*Quant, *BioTek *Instruments Inc., Winooski, VT, USA) at 570 nm and 690 nm (reference) wavelengths. The cell viability was calculated as percentage of viable cells in experimental group versus untreated control group using the following formula:

(1) $$\begin{array}{c} \text{cell}\;\text{viability}\;\;\left(\%\right)=\left(\frac{{\text{OD}}_{\exp}}{{\text{OD}}_{\text{control}}}\right)\times 100. \end{array}$$

The EC_50_ doses on HeLa cells were calculated from a graph of cell viability versus methanolic extract of *V. album* and nontoxic dose of the plant extract (10 *μ*g/mL) was used for further experiments. 

#### 2.4.1. Treatment of Extracts

HeLa cells (10^5^ cells/mL) were incubated with noncytotoxic doses of *V. album *extracts (10 *μ*g/mL) for experimental groups or solely with EMEM for the untreated control in the last 48 hours of the total incubation time of 72 hours. 

#### 2.4.2. Induction of Oxidative Stress

Oxidative stress was induced by H_2_O_2_ at 50 *μ*M and 100 *μ*M, and the depletion of H_2_O_2_ in the cell culture medium was measured. ROS generation was enhanced by treatment of the cells with 200 *μ*M H_2_O_2_. Harsh stress conditions to induce DNA damage were created by 750 *μ*M H_2_O_2_. After treatment with the extracts, the cells were washed with PBS and then treated with H_2_O_2_ for 1 h. Hank's balanced salt solution (HBSS) was used to dilute the H_2_O_2_ and as a blank in the assays. H_2_O_2_ concentration was checked by absorbance at 240 nm, as described by Aebi [17].

Depletion of H_2_O_2_ in culture media was measured at 10, 30 and 60 min as indicated in Figure 2 by the colorimetric method of Pick and Keisari [18]. In this assay H_2_O_2_ is reduced while phenol red is oxidized by the action of HRP. The final purple product exhibits maximum absorption at 612 nm. Briefly, the cells (10^5^ cells/mL) were seeded into each well, containing 200 *μ*L EMEM in a 96-well plate. Cell-free (negative) controls were run in parallel with the experimental groups. The cell culture medium (100 *μ*L) containing HBSS plus 50 *μ*M and 100 *μ*M H_2_O_2_ was thoroughly mixed with 200 *μ*L phenol red/horseradish peroxidase solution. The mixture was incubated at 37°C for 5 min, and the reaction was terminated by adding 2 *μ*L of 1 N NaOH. H_2_O_2_ concentrations were calculated using a standard curve of H_2_O_2_ versus absorbance (610 nm). The standard curve showed a linear relationship between absorbance at 610 nm and H_2_O_2_ concentration in the 12.5–100 *μ*M range. 

#### 2.4.3. Intracellular ROS Level

Intracellular ROS level was estimated by using a fluorescent probe, 2′,7′-dichlorofluorescein diacetate (DCFH-DA) [19]. After incubation and exposure to oxidative stress, the culture medium was immediately removed, and the cells were washed with PBS followed by incubation for 15 min in 10 *μ*M DCFH-DA (100 *μ*L) at 37°C in an atmosphere with 5% CO_2_. The fluorescence of hydrolyzed 2,7-dichlorofluorescein (DCF) was measured at 10 min intervals for 1 h in a microplate fluorometer with 485 nm excitation/530 nm emission wavelengths (FLx800, BioTek Instruments Inc., Winooski, VT, USA). The relative percentage of ROS production was calculated according to the following equation:

(2) $$\begin{array}{c} \text{ROS}\;\left(\%\right)=\left(\frac{F_{1}}{F_{0}}\right)\times 100\%, \end{array}$$

where F_0_ is fluorescence intensity of untreated control group and F_1_ is fluorescence intensity of experimental group.

### 2.5. DNA Isolation

High molecular weight total cellular DNA was isolated with the GenElute Mammalian Genomic DNA Isolation kit (Sigma, St. Louis, MO), according to the manufacturer's protocol. The concentration of total cellular DNA was determined by using the Quant-iT dsDNA High Sensitivity Assay Kit in a Qubit fluorometer (Invitrogen, Paisley, UK). 

### 2.6. Quantitative Polymerase Chain Reaction (QPCR)

QPCR was carried out according to the method of Erol et al. [20]. Three different genomic regions were analyzed for oxidative damages. Oligonucleotide primers specific for a 2082 bp fragment of the nuclear APEX1 gene (transcribed,GenBank ID: X66133) [21], a 2334 bp fragment of the nuclear *β*-globin gene cluster (nontranscribed, GenBank ID: NG_000007), and a 2232 bp fragment of the mtDNA **(**GenBank ID: AF347015) were used in the PCR reaction [20]. All PCR reactions were performed in Techne TC-3000 Thermocycler (Techne Inc., USA). The total volume of the reaction mixture was 50 *μ*L, containing 100 ng of DNA, 1x buffer (Fermentas), 0.2 mM dNTPs, 1–2.5 mM MgCl_2_, 0.3 *μ*M primers, and 2.5 unit of recombinant Taq DNA polymerase (Fermentas). A quantitative control using half of the concentration of control template DNA was included in each set of PCR reaction. The small fragments, 161 bp for mitochondrial DNA and 181 bp for nuclear region, were also amplified as internal controls to normalize the results obtained with the large fragments and to monitor mitochondrial copy number. An aliquot of each PCR product was checked by electrophoresis on a 1-2% (w/v) vertical agarose gel at 70 V for 45 min in 1xTAE buffer (Tris-acetate-EDTA, pH 8.0). The amount of QPCR products was quantified using Quant-iT dsDNA High Sensitivity Assay Kit. The average lesion frequency per each fragment was calculated using the *Poisson* equation [22].

### 2.7. Statistical Analysis

Data were expressed as mean ± SEM of three independent experiments done in triplicates. All analyses were carried out using GraphPad Prism, version 5.00 for Windows, GraphPad software Inc., San Diego, CA, http://www.graphpad.com/. Statistical analyses were performed using one-way ANOVA followed by Dunnett's test. Differences between two groups relation to the depletion kinetics of H_2_O_2_ were evaluated by using the two-tailed unpaired *t*-test. The probability values of *P* < 0.05 were considered as significant.

## 3. Results 

**Figure 1:**
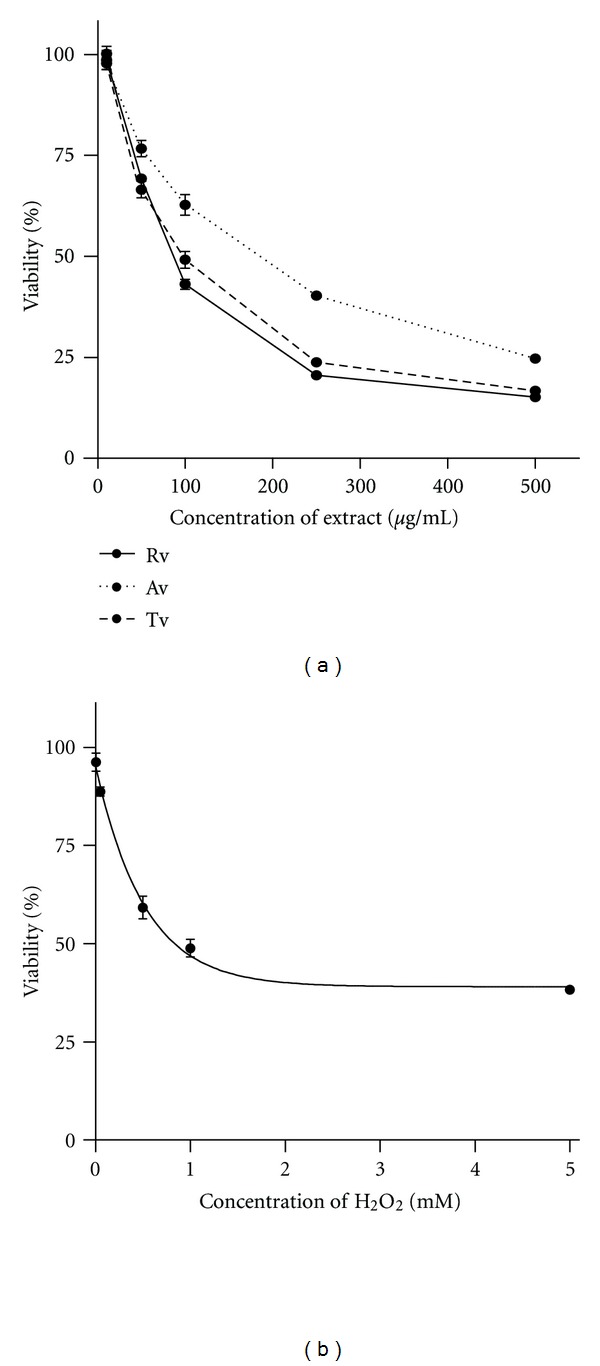
(a) Effect of methanolic extracts of *Viscum album* from different hosts on viability of HeLa cells, (b) cytotoxic effect of H_2_O_2_.

**Figure 2:**
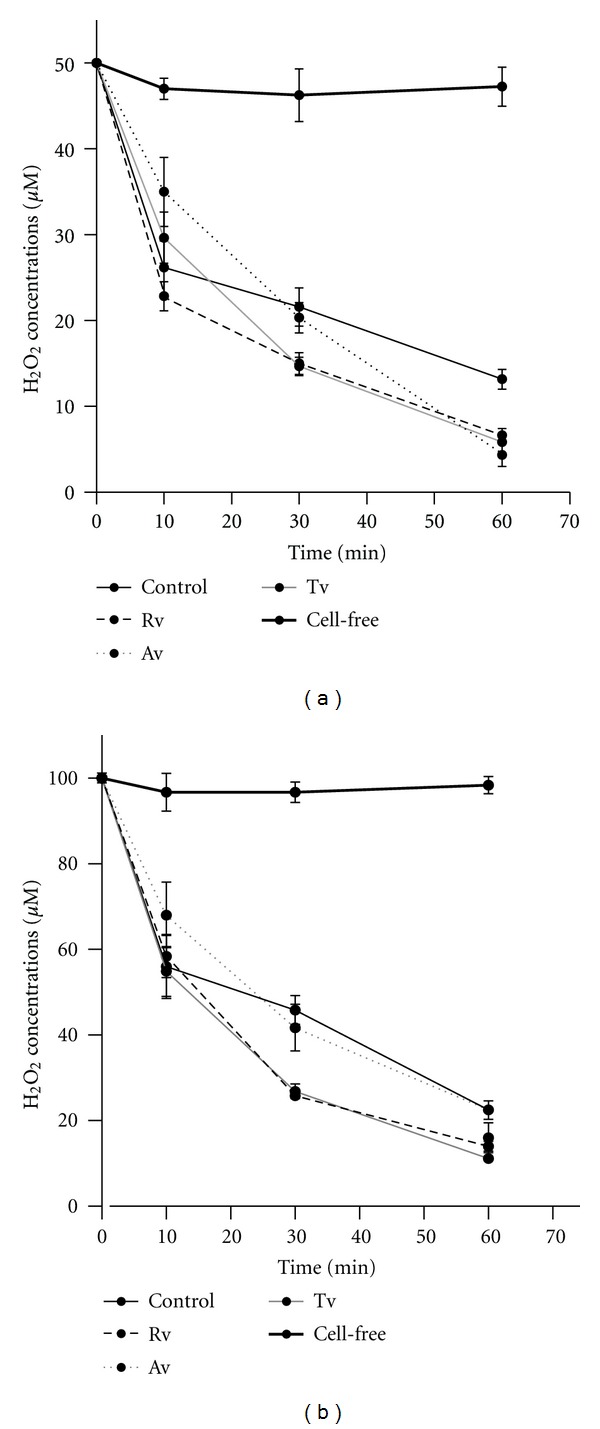
Effect of pretreatment with *Viscum album* extracts on depletion kinetics of (a) 50 *μ*M and (b) 100 *μ*M H_2_O_2_ in culture media.

All extracts and H_2_O_2_ decreased the viability of HeLa cells in a dose-dependent manner (Figure 1). As illustrated in Figure 1, the half maximal inhibitory concentration (IC_50_) of Tv, Av, Rv, and H_2_O_2_ is 93 *μ*g/mL, 165 *μ*g/mL, 85 *μ*g/mL, and 810 *μ*M, respectively. The noncytotoxic doses of all extracts (10 *μ*g/mL) were used for further experiments in order to avoid cell death as a result of cytotoxicity. Oxidative stress was provoked by 50 *μ*M and 100 *μ*M H_2_O_2_, and its depletion within 60 min was checked by monitoring the remaining H_2_O_2_ in the culture media. There were no significant differences between the depletion kinetics of both 50 *μ*M and 100 *μ*M H_2_O_2_ of cells treated with extracts (Figure 2). Almost 80% of H_2_O_2_ in culture media were depleted by HeLa cells within 1 hour, while H_2_O_2_ level in cell-free culture medium remained stable. These results indicated that H_2_O_2_ did not interact with the components of medium, and depletion of H_2_O_2_ occurred via cellular metabolism [23]. 

The incubation of the HeLa cells with 200 *μ*M H_2_O_2_ increased the level of ROS. Intracellular ROS production was increased by 19.2% in H_2_O_2_-treated cells compared with untreated control cells (Table 1). *V. album* methanolic extracts have no effect on the steady-state level of intracellular ROS. Av had the highest ROS inhibiting activity compared to 200 *μ*M H_2_O_2_-treated cells. Also, other two extracts (Tv and Rv) were found to have significant inhibitory effects on ROS generation following the oxidative stress induction (Table 1) by using gallic acid (GA) as reference substance.

**Figure 3:**
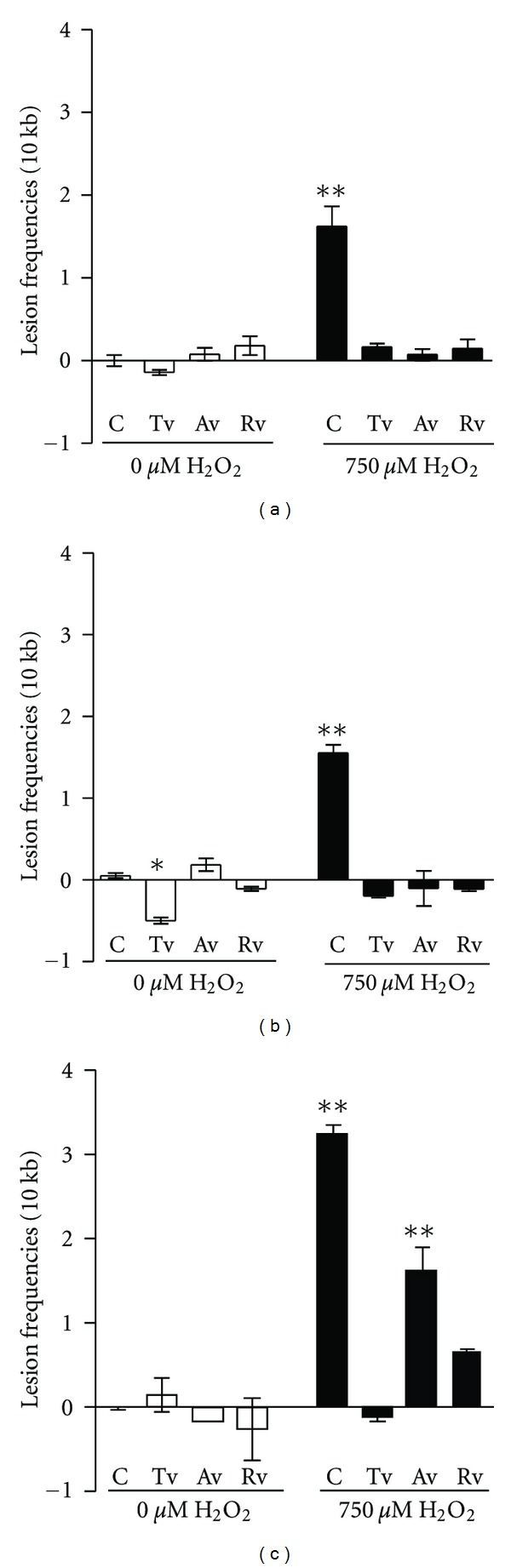
Effect of *Viscum album* extracts and/or H_2_O_2_ on (a) APEX1 gene; (b) *β*-globin region; (c) mtDNA in HeLa cells. C: Untreated control cells; Tv, Av, and Rv: Cells treated with 10 *μ*g/ml of the corresponding extract for 48 h. **P < 0.01; mean value were significantly different from the untreated control.

The gene-specific QPCR assay was performed on nuclear regions (APEX1 and *β*-globin) and mitochondrial DNA for the detection of oxidative damage caused by H_2_O_2_. The assay is based on the fact that many DNA lesions can block the Taq polymerase, and as a result, the product amplification is decreased [24]. When the different H_2_O_2_ concentrations (300 and 750 *μ*M) were applied to determine lesion frequencies in HeLa cells, it was found that in APEX1 and mtDNA regions were similarly induced by 300 *μ*M H_2_O_2_, while in *β*-globin region no significant induction was seen relative to control (unpublished data). Therefore, to determine the protective effect of the plant extract on cells, only 750 *μ*M H_2_O_2_ has been applied. In this study, lesion numbers per 10 kb on both nuclear and mitochondrial DNA in HeLa cells incubated with or without *Viscum* extracts were very low under nonstressed conditions (Figure 3). A negative value obtained for Tv in the *β*-globin region indicated that lesion frequency was much lower than in the others (*P* < 0.05). However considerable damage in all regions was detected, after the control cells were treated by 750 *μ*M H_2_O_2_. Lesion frequency of mtDNA was higher 2 times than nDNA. As expected, mtDNA was the most sensitive to oxidative stress induced by 750 *μ*M H_2_O_2_. It contained 3.2 lesions per 10 kb fragment, while transcribed (APEX1) and nontranscribed (*β*-globin) nDNAs were less damaged (1.6 and 1.7 lesions per 10 kb, resp.). In the experimental groups the methanolic extracts of *Viscum album* grown on different host trees exhibited different degrees of protective effect against oxidative DNA damage. Pretreatment with Rv and Tv extracts completely prevented the damage on both nuclear and mitochondrial DNA under stress conditions. However Av showed only ~50% inhibition against mtDNA damage, while it was entirely effective against nDNA damage.

## 4. Discussion 

Oxidative stress is an imbalance between formation of ROS and antioxidant defense, resulting in potential cellular damage [3]. Aging and several diseases such as cancers, diabetes, and degenerative disorders have been linked to oxidative stress caused by ROS [1, 25]. In living cells ROS are the primary source of oxidative DNA damage under both physiological and increased oxidative stress conditions [25]. Eukaryotic cells protect themselves by antioxidant defence mechanisms such as enzymes, radical scavengers, hydrogen donors, electron donors, peroxide decomposers, singlet oxygen quenchers, enzyme inhibitors, synergists, and metal-chelating agents [26]. Antioxidants may also repair oxidative damage or enhance antioxidant defense either by induction of phase II enzymes or by stimulating mitochondrial biogenesis [27]. Hence, DNA, proteins, and lipid constituents of the cells are protected by antioxidants against oxidative damage. Plants contain various antioxidant constituents that act in different ways and are used in traditional medicine. Depending on the bioactivity, plant extracts as well as many compounds isolated from them are extensively used in pharmaceutical industry as an ingredient drugs or cosmetics; some of these are able to protect against molecular damage [28]. For example, various phenolics compounds and antioxidants reduce oxidative DNA damage in different cell types [20, 29, 30]. 

The purpose of this study was to evaluate the protective effect of *Viscum album* methanolic extracts against nuclear and mitochondrial DNA damage in HeLa cells. *In vitro* antioxidant activity of this extract has been reported earlier [12, 13]. In our previous study composition and activity of *Viscum album* methanolic extract are found to be dependent on the host tree as well as time of harvesting [12]. In this present study we first showed that *Viscum album* methanolic extracts are able to inhibit ROS formation induced by H_2_O_2_ in HeLa cells. Similar results were obtained in an animal study that reported protective effect of *Viscum album* against oxidative stress on kidney and heart of diabetic rats [11]. Hence, it is suggested that *V. album* may protect living cells by inhibition of ROS formation. Surprisingly, the experimental dose of *V. album* (10 *μ*g/mL) used in the assay did not alter the level of basal damage of both nDNA and mtDNA when there was no induction of stress (Figure 3). This result may show that undetectable damage has occurred under physiological conditions. We also found that the mitochondrial genome is significantly (*P* < 0.001) more sensitive to H_2_O_2_-induced oxidative stress than both nuclear loci (Figure 3). Previous studies demonstrated that mtDNA is more susceptible to oxidative agents than nDNA [23, 31]. The susceptibility of mtDNA to oxidative damage is due to chain-propagation reactions or electron leakage from the respiratory chain [32]. mtDNA does not have introns with a high transcription rate, providing a high probability of oxidative modification of the expressed region [33]. Also the presence of localized metal ions in mitochondria may function as catalysts for the generation of ROS and the stimulation of secondary ROS reactions due to damage to the ETC and/or through lipid peroxidation [23].

Furthermore, we demonstrate that *Viscum album* methanolic extracts are able to prevent DNA damage induced by H_2_O_2_. It is predicted that several thousand DNA lesions per cell occur each day in humans by different stress factors [34]. If the cells do not have effective DNA repair and/or antioxidant defense mechanisms, this damage accumulates in DNA (in especially mtDNA), and this may result in age-related disorders. DNA damage in tumor suppressor genes and oncogenes may also be a likely cause of cancer [3–5]. Therefore it is especially significant that *Viscum album* can protect mtDNA, which has an important role in the aging and APEX1 gene (actively transcribed for a DNA repair enzyme) against this accumulation via inhibition of oxidative DNA damage. Nevertheless, no significant correlation between prevention of DNA damage and the inhibition of ROS was obtained, suggesting that the antioxidant potential of *Viscum album* extract is generated by a complex synergy and different mechanisms of active molecules, not only by ROS inhibition.

Antioxidant activity of plants is associated with their bioactive compounds, mainly antioxidant phenolics, because of their ability to scavenge free radicals. In *V. album* methanolic extracts, the total quercetin content was measured as 4.92 *μ*g/g crude extract of Tv, 3.92 *μ*g/g crude extract of Av, 7.97 *μ*g/g crude extract of Rv, respectively (unpublished data). Thus, the high quercetin content in *V. album* extract may be responsible for the protection of DNA damage in HeLa cells.

In conclusion, taken together these results suggest that methanolic extract of *Viscum album* can protect against DNA damage via direct inhibition of ROS formation and/or indirectly by other mechanisms, through induction of oxidative damage repair and phase II enzymes. Therefore, dietary intake of *Viscum album* extract may lower the risk of oxidative stress-mediated diseases such as some cancers, diabetic complications, degenerative and gastrointestinal diseases, or atherosclerosis via reduction of oxidative DNA damage and intracellular levels of ROS. Further studies are needed in order to define the possible beneficial outcomes of its dietary use and to identify mechanisms of action.

## Figures and Tables

**Table 1:** Effect of pretreatment with *Viscum album* from different hosts on intracellular ROS generation.

	Relative amount of intracellular ROS (%)	
	No stress induction (0 *μ*M H_2_O_2 _)	Stress induction (200 *μ*M H_2_O_2 _)
Untreated control	100.00 ± 0.41	119.20 ± 1.72**
GA (28 *μ*g/mL)	96.70 ± 3.33	102.20 ± 2.46^●●^
Tv (10 *μ*g/mL)	97.36 ± 2.40	108.63 ± 1.64^●^
Av (10 *μ*g/mL)	99.49 ± 2.65	101.10 ± 2.26^●●^
Rv (10 *μ*g/mL)	102.70 ± 1.95	108.61 ± 2.45^●^

*n* = 6, **P < 0.01 in comparison with untreated control, ^●^ *P* < 0.05, ^●●^ *P* < 0.01 in comparison with 200 *μ*M H_2_O_2_ (GA: Gallic acid, Tv: *Tilia viscum*, Av: *Acer viscum*, Rv: *Robinia viscum*).

## Abbreviations

Av: Viscum album grown on Acer campestre
GA: Gallic acid
GAE: Gallic acid equivalent
HBSS: Hank's balanced salt solution
mtDNA: Mitochondrial DNA
nDNA: Nuclear DNA
QPCR: Quantitative PCR
Rv: *Viscum album* grown on *Robinia pseudoacacia*
Tv: *Viscum album* grown on *Tilia argentea*.
